# Research on Open Magnetic Shielding Packaging for STT and SOT-MRAM

**DOI:** 10.3390/mi16101157

**Published:** 2025-10-13

**Authors:** Haibo Ye, Xiaofei Zhang, Nannan Lu, Jiawei Li, Jun Jia, Guilin Zhao, Jiejie Sun, Lei Zhang, Chao Wang

**Affiliations:** 1 The 58th Research Institute of China Electronics Technology Group Corporation, Wuxi 214000, China; d201780197@alumni.hust.edu.cn (H.Y.); 18649868330@163.com (N.L.); ljw97119@126.com (J.L.); jun.jia.work@outlook.com (J.J.); htron@foxmail.com (G.Z.); sunjiejie1989@126.com (J.S.); 13603214005@163.com (L.Z.); 2 National Innovation Institute of Defense Technology, Beijing 100071, China; zxf1990fei@163.com

**Keywords:** magnetic random access memory, spin-transfer torque, anti-magnetic field interference

## Abstract

As an emerging type of non-volatile memory, magneto-resistive random access memory (MRAM) stands out for its exceptional reliability and rapid read–write speeds, thereby garnering considerable attention within the industry. The memory cell architecture of MRAM is centered around the magnetic tunnel junction (MTJ), which, however, is prone to interference from external magnetic fields—a limitation that restricts its application in demanding environments. To address this challenge, we propose an innovative open magnetic shielding structure. This design demonstrates remarkable shielding efficacy against both in-plane and perpendicular magnetic fields, effectively catering to the magnetic shielding demands of both spin-transfer torque (STT) and spin–orbit torque (SOT) MRAM. Finite element magnetic simulations reveal that when subjected to an in-plane magnetic field of 40 mT, the magnetic field intensity at the chip level is reduced to nearly 1‰ of its original value. Similarly, under a perpendicular magnetic field of 40 mT, the magnetic field at the chip is reduced to 2‰ of its initial strength. Such reductions significantly enhance the anti-magnetic capabilities of MRAM. Moreover, the magnetic shielding performance remains unaffected by the height of the packaging structure, ensuring compatibility with various chip stack packaging requirements across different layers. The research presented in this paper holds immense significance for the realization of highly reliable magnetic shielding packaging solutions for MRAM.

## 1. Introduction

Magnetic random access memory (MRAM) has garnered widespread attention owing to its exceptional attributes, including non-volatility, rapid read–write speeds, an almost infinite number of read–write cycles, and low power consumption [1,2,3,4,5,6]. As MRAM employs magnetism for data recording, it is prone to interference from external magnetic fields, which can result in read–write errors [7,8,9]. Consequently, implementing a magnetic shielding packaging structure to mitigate the impact of external magnetic fields on the memory holds significant importance [10,11,12].

Currently, the predominant MRAM technologies are based on spin-transfer torque (STT) [13] and spin–orbit torque (SOT) [14]. STT-MRAM exhibits heightened sensitivity to magnetic fields perpendicular to the plane, whereas SOT-MRAM is more susceptible to magnetic fields parallel to the plane [15]. To address the in-plane sensitivity of SOT-MRAM, utilizing two parallel magnetic shielding plates can effectively shield against external magnetic fields [16]. This approach is straightforward to implement, offers high reliability, and does not necessitate special treatment of the packaging leads; however, it provides no shielding against perpendicular magnetic fields [17]. In contrast, to counter the perpendicular magnetic field sensitivity of STT-MRAM, current packaging technologies predominantly employ a can-type structure. Although this structure can shield against perpendicular magnetic fields, it demands stringent process requirements, suffers from low yield rates, and experiences magnetic leakage at the lead openings, potentially leading to read–write errors in chips located near these openings [18,19].

In response to the aforementioned challenges, this work introduces an open magnetic shielding packaging structure that is compatible with both perpendicular STT and in-plane SOT MRAM. This innovative structure effectively shields against magnetic fields in both perpendicular and in-plane directions. Moreover, it features a simple design, offers high reliability, and accommodates stacked packaging requirements, thereby presenting vast potential for future applications.

## 2. Methodology

Numerical simulations of the static magnetic field distribution were performed using the “Magnetic Fields, No Currents” 3D module in COMSOL Multiphysics (version 6.0). The shielding plates were modeled as permalloy (1J79) with a nickel content of 77%. The magnetic properties of permalloy adopt the B-H curve built into the software. The chip is composed of silicon, and its relative permeability is basically the same as that of air. Therefore, its influence on the magnetic field is not considered.

## 3. Results and Discussion

The magnetic field environment surrounding MRAM is typically composed of low-frequency varying magnetic fields, which permits approximate analysis of the magnetic shielding effectiveness of the shielding structure for the memory using a constant magnetic field model. Initially, the principle of magnetic shielding is qualitatively analyzed through the concepts of magnetic circuits and magnetic reluctance. Just as electrical dielectrics are characterized by dielectric permittivity, magnetic media can be characterized using magnetic reluctance, which helps describe the effect of the magnetic circuit on the magnetic field. Within a magnetic circuit, magnetic flux tends to flow through paths with low magnetic reluctance. Therefore, by constructing low-reluctance paths around the chip, the magnetic field can be channeled and diverted, thereby reducing the magnetic field intensity at the chip location.

For a small magnetic reluctance element within a magnetic circuit, where the magnetic field can be considered uniform, its magnetic reluctance *R_m_* is determined as follows:

(1) $$R_{m}=\frac{L}{\mu S}$$

where *μ* represents the magnetic permeability (H/m) of the material of the magnetic reluctance element, *S* is the cross-sectional area (m^2^) of the magnetic circuit, and *L* is the length (m) of the magnetic circuit. As indicated by the above formula, magnetic reluctance is inversely proportional to magnetic permeability; thus, a higher magnetic flux passes through materials with greater magnetic permeability.

In MRAM applications, soft magnetic materials are generally employed to fabricate the magnetic shielding package. Compared to air, soft magnetic materials possess significantly higher magnetic permeability, resulting in much lower magnetic reluctance. When an external magnetic field passes through the magnetic shielding structure surrounding the chip, the majority of the magnetic flux travels through the magnetic circuit composed of the soft magnetic material, while only a small amount of flux passes through the chip, thereby achieving the goal of magnetic field shielding.

**Figure 1:**
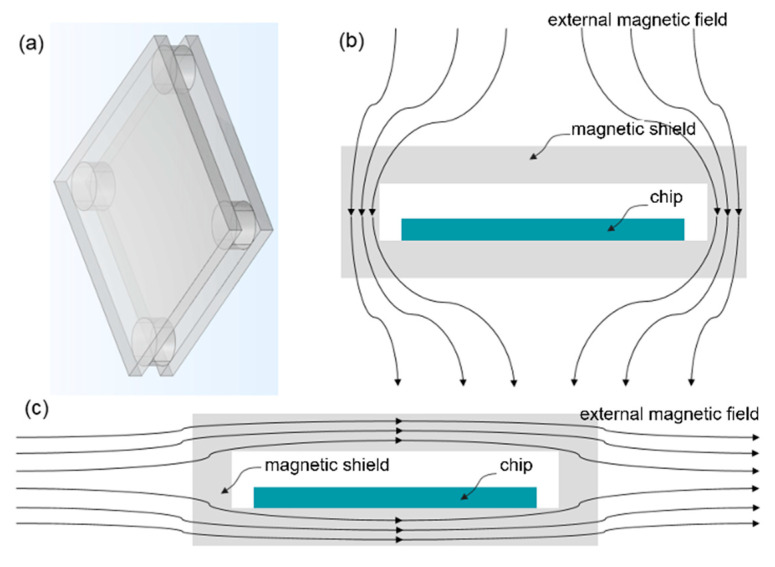
Schematic diagram of magnetic shielding principles. (**a**) Schematic diagram of the magnetic shielding structure. (**b**) Perpendicular magnetic field. (**c**) In-plane magnetic field.

Figure 1a presents a schematic of the magnetic shielding structure proposed in this paper, consisting of upper and lower magnetic shielding plates and four magnetic shielding cylinders. This structure provides magnetic shielding against both in-plane and perpendicular magnetic fields, making it suitable for SOT and STT-MRAM. The in-plane magnetic field refers to the magnetic field where its direction is parallel to the magnetic shielding plates plane, whereas a perpendicular magnetic field refers to the magnetic field where its direction is perpendicular to the magnetic shielding plates plane. The shielding principle is depicted in Figure 1b,c. Figure 1b,c are cross-sectional views of the magnetic shielding structure at the magnetic shielding cylinders. Figure 1b shows the scenario where the external magnetic field is perpendicular to the chip. In this case, the upper and lower magnetic shielding plates do not provide shielding because the magnetic flux entering the upper plate equals the flux exiting it, ultimately affecting the underlying chip. Instead, the magnetic shielding cylinders surrounding the chip are responsible for the shielding effect. The magnetic flux exiting the upper shielding plate is diverted by the surrounding cylinders before reaching the chip, thereby reducing the magnetic field intensity at the chip location. Notably, the magnetic shielding cylinders employed here feature an open structure, yet they still effectively shield the entire chip.

Figure 1c illustrates the situation when the external magnetic field is parallel to the chip. In this scenario, the surrounding magnetic shielding cylinders do not provide shielding because the magnetic flux entering and exiting the cylinders remains the same, still affecting the chip. Instead, the upper and lower magnetic shielding plates are responsible for the shielding effect, diverting the in-plane magnetic field and thereby reducing the magnetic field intensity at the chip.

From the aforementioned qualitative analysis, it can be seen that the upper and lower magnetic shielding layers and the surrounding magnetic shielding cylinders play roles in in-plane and perpendicular magnetic fields, respectively. Next, the finite element calculation method will be employed to quantitatively analyze the influencing factors of the magnetic shielding effectiveness. The theoretical basis for the finite element calculation is described by Maxwell’s equations for three-dimensional steady magnetic fields as follows:

(2) $$\nabla \times H\left(x,y,z\right)=J\left(x,y,z\right)$$

(3) $$\nabla \cdot B\left(x,y,z\right)=0$$

The 3D model utilizes permalloy (1J79) as the material, with the side length of the upper and lower magnetic shielding plates set at 0.01 m.

**Figure 2:**
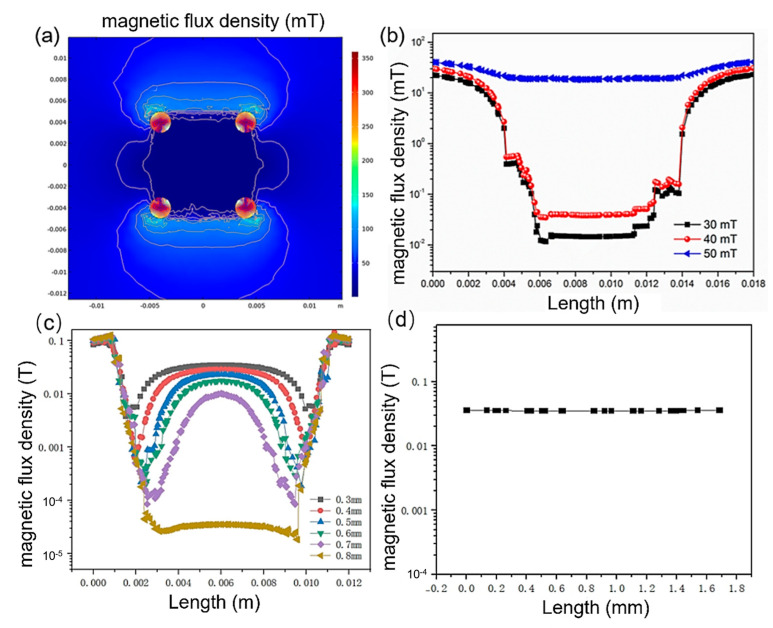
In-plane magnetic field shielding. (**a**) Internal magnetic field distribution. (**b**) Internal magnetic field distribution under external magnetic fields of 30, 40, and 50 mT. (**c**) Internal magnetic field distribution for different shielding plate thicknesses under a 50 mT external field. (**d**) Internal magnetic field distribution along the perpendicular direction of the shielding layers.

First, the shielding effectiveness of the magnetic shielding structure against in-plane magnetic fields has been analyzed. Figure 2a depicts the cross-sectional magnetic field distribution at the chip when the external magnetic field is parallel to it. As seen from the figure, the magnetic field distribution is highly uniform within the 0.01 × 0.01 m^2^ area and is significantly weaker than the external magnetic field strength, indicating good shielding effectiveness against in-plane magnetic fields within the internal space of the magnetic shielding plates. Figure 2b shows the magnetic field distribution at the chip plane when the external magnetic field strengths are 30 mT, 40 mT, and 50 mT. The horizontal axis “Length” in Figure 2b represents the position in Figure 1 that lies along the direction parallel to both the chip and the external magnetic field, and is situated at the center of the upper and lower magnetic shielding plates. The horizontal axis from 0.004 m to 0.014 m is covered by the magnetic shielding plates. When the external magnetic field is 30 mT and 40 mT, the magnetic field strength in this region sharply decreases to below 1 mT, demonstrating the shielding effectiveness of the area covered by the magnetic shielding plates. The magnetic field is notably weaker in the central region from 0.006 m to 0.012 m within the shielding structure, with the magnetic field decreasing to 0.02 mT at the center when the external field is 30 mT, representing a reduction to 0.67‰ of the original field strength, and to 0.04 mT when the external field is 40 mT, representing a reduction to 1‰ of the original field strength. Beyond the area from 0.06 m to 0.12 m, the magnetic field strength continues to weaken but remains below 0.6 mT. When the external magnetic field is further increased to 50 mT, a significant reduction in shielding effectiveness is observed, with the magnetic field inside the shielding structure becoming nearly identical to the external field. This occurs due to magnetic saturation. Although soft magnetic materials have low magnetic reluctance, allowing most of the magnetic flux to pass through, the maximum magnetic flux that can pass through a magnetic circuit with a fixed cross-sectional area does not increase linearly with the external magnetic field. When the external magnetic field strength increases to a certain level, the rate of increase in magnetic flux within the magnetic circuit slows down and gradually approaches saturation. An effective solution to magnetic flux saturation is to increase the cross-sectional area, such as by increasing the thickness of the magnetic shielding plates.

When the magnitude of the external magnetic field attains 50 mT, the shielding layers reach a state of magnetic saturation, rendering the shielding effect ineffective. This is evident from the 50 mT curve depicted in Figure 2b. To counteract the influence of stronger external magnetic fields, augmenting the thickness of the shielding layers represents an efficacious strategy. Figure 2c illustrates the internal magnetic field distribution under the influence of a 50 mT external magnetic field for shielding layers of varying thicknesses. For thicknesses ranging from 0.3 mm to 0.7 mm, the internal magnetic field exhibits non-uniformity: the field intensity at the edges is low, signifying robust shielding, whereas the central region displays a higher field intensity, indicating weaker shielding. As the thickness of the shielding layers increases, the intensity of the internal magnetic field gradually diminishes. For shielding layers of sufficient thickness, the magnetic field across the entire region is reduced to approximately 10^−4^ T with outstanding uniformity. Figure 2d presents the internal magnetic field distribution along the perpendicular direction of the shielding layers. The magnetic field distribution maintains a high degree of uniformity across different heights, thereby offering effective shielding for the chip at diverse positions.

**Figure 3:**
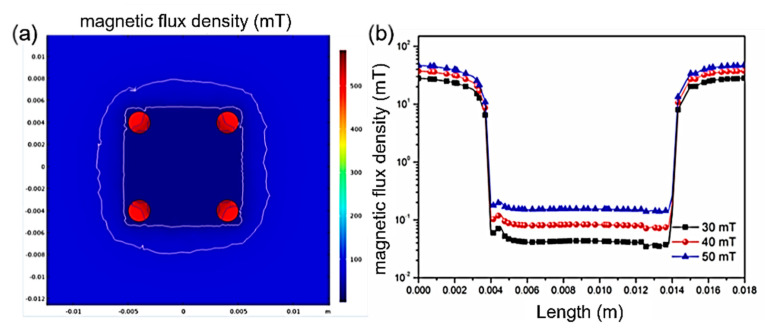
Shielding against perpendicular magnetic fields. (**a**) Internal magnetic field distribution. (**b**) Internal magnetic field distribution under external magnetic fields of 30, 40, and 50 mT.

The shielding effectiveness of the magnetic shielding structure against perpendicular magnetic fields has also been analyzed. Figure 3a presents the cross-sectional magnetic field distribution at the chip when the external magnetic field is perpendicular to it. As shown in the figure, the magnetic field distribution is remarkably uniform within the 0.01 × 0.01 m^2^ area and is significantly weaker than the external magnetic field intensity, indicating good shielding performance against perpendicular magnetic fields within the internal space of the magnetic shielding plates. Figure 3b displays the magnetic field distribution at the chip plane under external magnetic field strengths of 30, 40, and 50 mT. It can be observed that the magnetic field distribution remains highly uniform across the entire 0.01 × 0.01 m^2^ area, without any gradual changes in the edge regions. When the external field strength is 30 mT, the magnetic field intensity at the center is 0.04 mT, representing a reduction to 1.3‰ of the original strength. When the external field strength increases to 40 mT, the magnetic field intensity at the center is 0.08 mT, reduced to 2‰ of the original. When the external field strength reaches 50 mT, the magnetic field intensity at the center is 0.15 mT, decreased to 3‰ of the original. As the external magnetic field strength increases, the internal magnetic field intensity also rises gradually. Based on the above analysis, it can be concluded that the magnetic shielding structure proposed in this paper exhibits excellent shielding effectiveness against perpendicular magnetic fields, with a highly uniform magnetic field distribution across the entire shielding area. Therefore, the magnetic units located at the edges of the chip can also be effectively protected.

**Figure 4:**
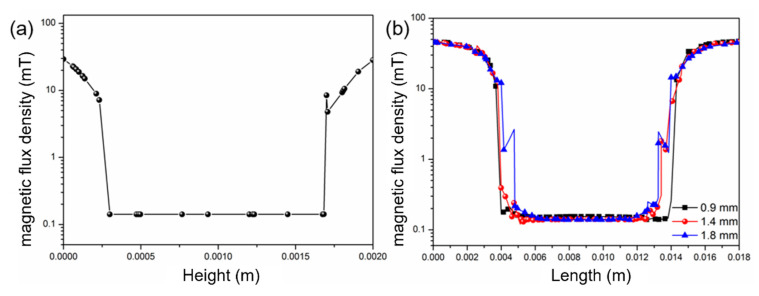
(**a**) Magnetic field distribution in the perpendicular direction. (**b**) Internal magnetic field distribution at different heights.

An effective approach to stacking memory chip capacities is through multi-layer laminated packaging. To meet this requirement, the magnetic shielding structure must ensure effective shielding of magnetic fields at different heights within its interior. Therefore, an analysis of the magnetic shielding effectiveness at various heights within this magnetic shielding structure is conducted. Figure 4a illustrates the magnetic field distribution perpendicular to the chip at the center of the shielding structure when a perpendicular magnetic field is applied. As depicted in the figure, there is good shielding effectiveness against perpendicular magnetic fields at different heights within the internal space of the magnetic shielding plates, with nearly identical magnetic fields at various heights. Figure 4b shows the internal magnetic field distribution at different heights of the magnetic shielding cylinders. From Figure 4b, it can be observed that when the heights of the magnetic shielding cylinders are 0.9, 1.4, and 1.8 mm, the internal magnetic field strengths are almost identical at the center of the shielding structure. When the height of the magnetic shielding cylinders is relatively high (1.8 mm), fluctuations in the magnetic field occur at the edge, 1 mm from the periphery, while the magnetic shielding effectiveness remains excellent in the internal region. Since PADs are generally distributed at the edges of the chip, the impact on magnetic units is minimal. Based on the above results, the magnetic shielding structure proposed in this work can accommodate different packaging heights while ensuring excellent shielding effectiveness, making it suitable for the stacked packaging requirements of MRAM with varying specifications.

**Figure 5:**
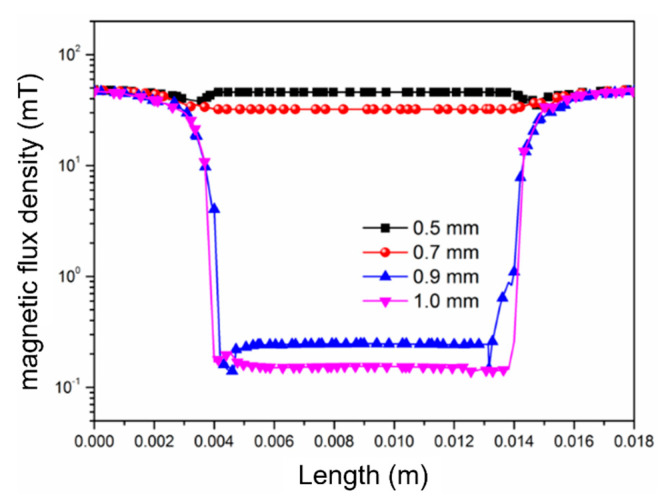
Internal magnetic field distribution for shielding cylinders with different radii.

Similar to in-plane magnetic fields, perpendicular magnetic fields can also exhibit magnetic saturation. Since the magnetic shielding cylinders are responsible for channeling the perpendicular magnetic field, the cross-sectional area of the cylinders perpendicular to the magnetic field direction is closely related to the magnetic saturation phenomenon. Figure 5 illustrates the internal magnetic field distribution within the shielding structure under different cross-sectional areas of the magnetic shielding cylinders when the external magnetic field strength is 50 mT. When the cylinder radius is 1.0 mm, the internal magnetic field is 0.15 mT, representing a reduction to 3‰ of the original strength. When the cylinder radius decreases to 0.9 mm, the internal magnetic field is approximately 0.25 mT, reduced to 5‰ of the original. From this trend, it can be observed that the cross-sectional area of the magnetic shielding cylinders significantly affects the magnetic shielding effectiveness. A larger cross-sectional area allows for a greater magnetic flux to pass through, resulting in better shielding performance. When the radius of the cylinders continues to decrease to 0.7 mm, the internal magnetic shielding effectiveness drops sharply. Further reduction in the radius to 0.5 mm leads to a further deterioration in the magnetic shielding effectiveness. Based on the above analysis, the cross-sectional area of the magnetic shielding cylinders determines the maximum external magnetic field strength that this structure can effectively shield. When the external magnetic field strength exceeds a critical value, the magnetic shielding cylinders reach magnetic flux saturation and can no longer channel additional magnetic flux. Consequently, this excess magnetic flux surges into the interior of the shielding structure. Therefore, when designing the shielding structure, it is essential to tailor the cross-sectional area of the magnetic shielding cylinders based on the limiting field strength of the application scenario.

Therefore, magnetic flux density saturation has a significant impact on magnetic shielding effectiveness. Special attention should be paid to the influence of flux density saturation under high magnetic fields when designing the parameters of the magnetic shielding structure. When the external magnetic field is in-plane, the thickness of the magnetic shielding plates is the primary consideration, and the thickness should be increased as much as possible under high magnetic fields. When the external magnetic field is perpendicular, the cross-sectional area of the magnetic shielding cylinders becomes the primary consideration, and the cross-sectional area should be increased as much as possible under high magnetic fields.

**Figure 6:**
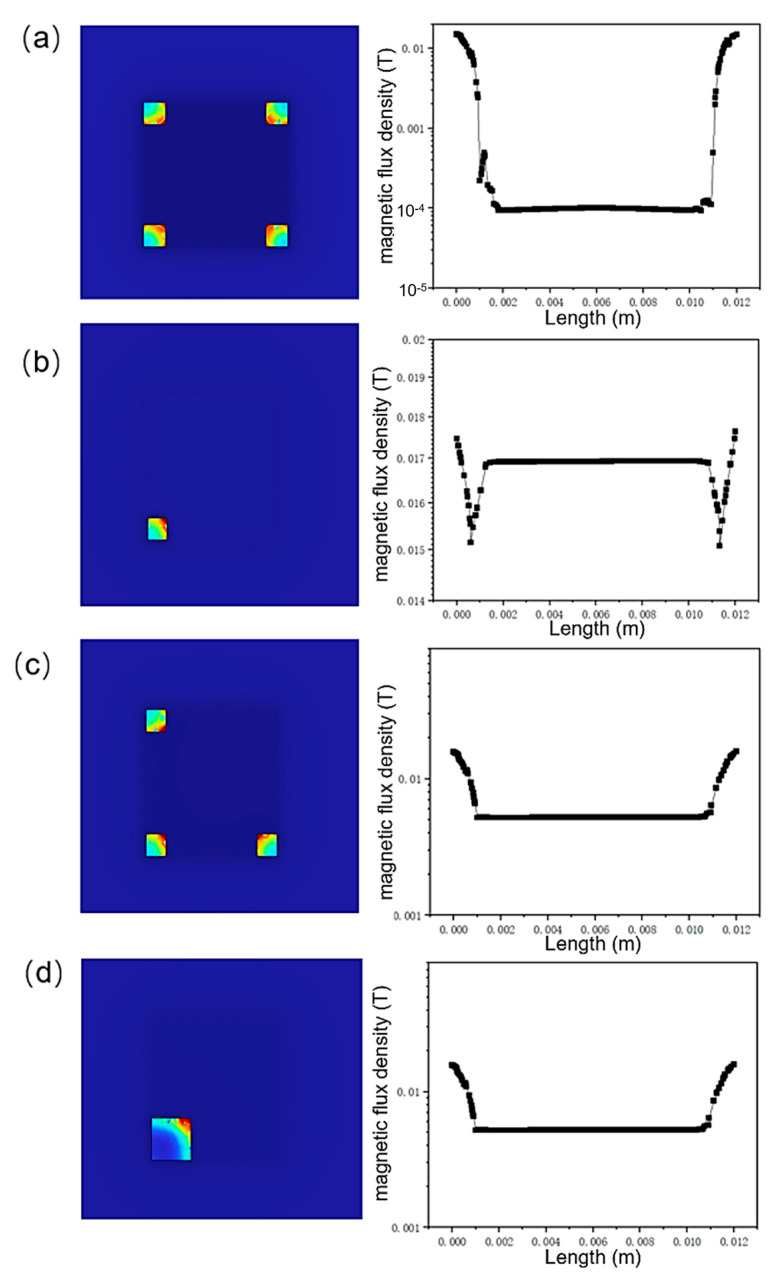
The Influence of magnetic shielding cylinders on magnetic field distribution. (**a**) Four cylinders; (**b**) One cylinder; (**c**) Three cylinders; (**d**) One cylinder with larger cross-sectional area.

In the magnetic shielding structure under consideration, shielding cylinders are designated as the key components responsible for mitigating the influence of perpendicular magnetic fields. To conduct a more comprehensive assessment of their impact, an in-depth analysis was carried out to examine the effects of cylinder distribution, shape, and cross-sectional area on the overall shielding performance. As illustrated in Figure 6a, when the shielding cylinders are square in shape, the magnetic shielding structure still demonstrates outstanding shielding capabilities. Specifically, the magnetic field within the plate is attenuated to a level of 10^−4^ T. This result implies that the shape of the cylinders has a negligible influence on the shielding effect. Consequently, this characteristic significantly enhances the manufacturability of the proposed structure, as it relaxes the constraints on the cylinder shape during the manufacturing process. Figure 6b presents a scenario where only a single shielding cylinder is employed. In this case, the internal magnetic field reaches a relatively high value of 0.017 T, indicating a rather poor shielding performance. When the number of cylinders is increased to three, as depicted in Figure 6c, the internal magnetic field decreases to 0.004 T. However, this performance is still inferior to that of the structure with four cylinders, highlighting the positive correlation between the number of cylinders and the shielding effectiveness to a certain extent. Figure 6d showcases the shielding effect when a single cylinder with a cross-sectional area four times larger than the original is used. Although the internal magnetic field is reduced to 0.004 T, this performance is markedly worse compared to the case shown in Figure 6a. This comparison clearly demonstrates that the spatial distribution of the shielding cylinders plays a pivotal role in achieving effective magnetic shielding. It is not merely the number or the cross-sectional area of the cylinders that matters, but rather their strategic arrangement within the structure that determines the overall shielding performance.

**Figure 7:**
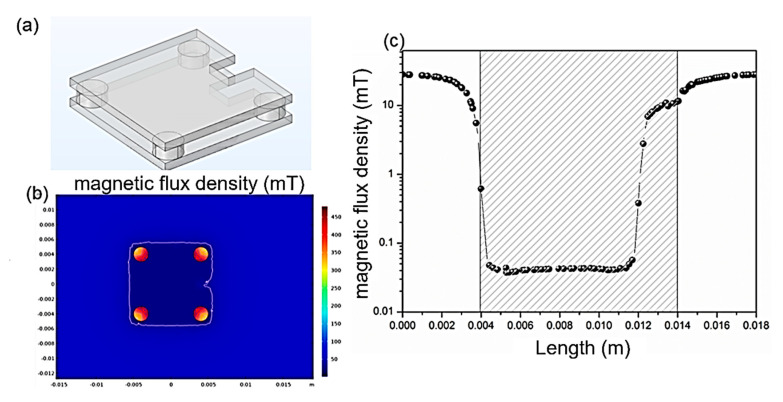
Effect of notches in the magnetic shielding plate on the magnetic field distribution. (**a**) The 3D model of the shielding structure with notches; (**b**) The internal magnetic field distribution; (**c**) The linear distribution of the internal magnetic field (The shaded areas correspond to the position of the magnetic shielding plates).

The proposed structure adopts an open-type configuration, which is specifically engineered to deliver effective shielding against perpendicular magnetic fields across the entire region encompassed by the magnetic shielding plates. In this design paradigm, the shielding cylinders predominantly function as flux-guiding elements. It has been experimentally and theoretically verified that increasing the cross-sectional area of these cylinders significantly enhances the overall shielding capacity of the entire package. To delve deeper into the function of the shielding layers that are parallel to the chip when subjected to perpendicular magnetic fields, notches were deliberately introduced into the structure. In the simulation model, as depicted in Figure 7a, rectangular blocks were excised from both the upper and lower shielding plates. Subsequently, the modified structure was exposed to a perpendicular magnetic field. Figure 7b clearly shows that in the areas still covered by the shielding plates, the magnetic field remains effectively shielded, maintaining a low-field state. However, near the notches, concave distortions are observable in the magnetic equipotential lines. These distortions are indicative of a locally elevated magnetic field intensity, suggesting a disruption in the normal shielding mechanism. Figure 7c further corroborates this observation. At the precise location of the notches (0.012 mm), there is a substantial increase in the internal field strength. This significant rise in field intensity clearly demonstrates a loss of shielding effectiveness against perpendicular magnetic fields in the vicinity of the notches. The computational results obtained from this study unveil a crucial aspect of the shielding mechanism under perpendicular magnetic fields. The shielding plates parallel to the chip serve as flux pathways, directing the magnetic flux into the shielding cylinders. In regions where notches are present, there is an absence of a low-resistance magnetic path. As a result, the perpendicular magnetic field can pass directly through the air and into the chip, thereby degrading the shielding performance. Moreover, this analysis also implies that the thickness of the shielding plates imposes a limit on the maximum shielding capacity when dealing with perpendicular magnetic fields. Once the shielding plates reach a state of magnetic saturation, they lose their ability to effectively guide the magnetic flux into the cylinders. Consequently, the excess magnetic flux bypasses the shielding mechanism and passes directly into the chip. In such scenarios, increasing the thickness of the shielding plates may present an effective solution to enhance the shielding performance and prevent magnetic saturation.

The core of the structure proposed in this paper lies in two magnetic shielding plates and four magnetic shielding cylinders connecting these plates. As an open structure, it enables heat dissipation from all four sides, thus exhibiting excellent heat dissipation performance and making it applicable even for high-power MRAM chips. The magnetic shielding effectiveness of this structure is independent of the shape of the cylinders. Therefore, the shape of the cylinders can be designed to reduce processing difficulty (e.g., designing a shape suitable for milling). Since this structure is relatively simple, it offers certain cost advantages compared with the hexahedral magnetic shielding structure.

## 4. Conclusions

In summary, this work presents an open magnetic shielding structure and a corresponding design guideline that effectively shields against magnetic fields in both in-plane and perpendicular directions, substantially reducing the magnetic field intensity at the chip. When the in-plane magnetic field is 40 mT, the magnetic field at the chip is reduced to 1‰ of its original value; when the perpendicular magnetic field is 40 mT, it is reduced to 2‰ of the original value. Furthermore, by adjusting the height of the cylindrical component, the structure can accommodate the stacking requirements of chips with different layer counts without compromising the shielding performance. Table 1 shows a comparison between this work and other relevant results. The structure in this work can cover the shielding requirements of STT and SOT-MRAM. Given that the bonding wires of the PADs can be led out from the four side faces, there is no need to modify the MRAM PAD layout in the proposed dihedral open magnetic shielding structure. When the thickness of shielding layers is 0.8 mm and the cylinder radius is 1.0 mm, the proposed structure achieves a 2‰ shielding effectiveness, which is one order of magnitude lower than other reported results. The research in this paper offers a novel approach to magnetic shielding design for MRAM.

## Figures and Tables

**Table 1:** Performance comparison with existing magnetic shielding structures for MRAM.

Shielding Structure	Suitable Chip Types	Need for PAD Layout Modification	Shielding Effectiveness
Trihedral structure [17]	STT-MRAM	Yes	16%
Hexahedral structure [18]	STT-MRAM	Yes	5%
Hexahedral structure [11]	STT-MRAM	Yes	20%
Dihedral structure	SOT-MRAM	No	/
Dihedral structure with four cylinders (this work)	Both for STT-MRAM and SOT-MRAM	No	2‰

## Data Availability

All data needed to evaluate the conclusions in the paper are present in the paper.
